# Expression of Heat Shock Proteins in Thermally Challenged Pacific Abalone *Haliotis discus hannai*

**DOI:** 10.3390/genes11010022

**Published:** 2019-12-23

**Authors:** Dongsoo Kyeong, Juyeon Kim, Younhee Shin, Sathiyamoorthy Subramaniyam, Byeong-Chul Kang, Eun-Ha Shin, Eun Hee Park, Eun Soo Noh, Young-Ok Kim, Jung Youn Park, Bo-Hye Nam

**Affiliations:** 1 Research and Development Center, Insilicogen Inc., Yongin-si, Gyeonggi-do 16954, Korea; dskyeong@insilicogen.com (D.K.); juykim@insilicogen.com (J.K.); yhshin@insilicogen.com (Y.S.); moorthy@insilicogen.com (S.S.); bckang@insilicogen.com (B.-C.K.); 2 Laboratory of Developmental Biology and Genomics, Seoul National University, Seoul 08826, Korea; 3 Department of Biological Sciences, Sungkyunkwan University, Suwon 16419, Korea; 4 Biotechnology Research Division, National Institute of Fisheries Science, Busan 46083, Korea; 2110018@nifs.go.kr (E.-H.S.); 549@nifs.go.kr (E.H.P.); laperm@korea.kr (E.S.N.); yobest12@korea.kr (Y.-O.K.); genome@korea.kr (J.Y.P.)

**Keywords:** cold, heat shock proteins, pacific abalone, transcriptome, thermal

## Abstract

Summer mortality, caused by thermal conditions, is the biggest threat to abalone aquaculture production industries. Various measures have been taken to mitigate this issue by adjusting the environment; however, the cellular processes of Pacific abalone (*Haliotis discus hannai*) have been overlooked due to the paucity of genetic information. The draft genome of *H. discus hannai* has recently been reported, prompting exploration of the genes responsible for thermal regulation in Pacific abalone. In this study, 413 proteins were systematically annotated as members of the heat shock protein (HSP) super families, and among them 26 HSP genes from four Pacific abalone tissues (hemocytes, gill, mantle, and muscle) were differentially expressed under cold and heat stress conditions. The co-expression network revealed that HSP expression patterns were tissue-specific and similar to those of other shellfish inhabiting intertidal zones. Finally, representative HSPs were selected at random and their expression patterns were identified by RNA sequencing and validated by qRT-PCR to assess expression significance. The HSPs expressed in hemocytes were highly similar in both analyses, suggesting that hemocytes could be more reliable samples for validating thermal condition markers compared to other tissues.

## 1. Introduction

Abalone is a shellfish belonging to the family Haliotidae (class: Gastropoda). Its growth and development are influenced by various environmental factors, such as temperature, oxygen, CO_2_, and salinity. Among these, temperature is the most important factor, having a higher correlative effect with other stressors as well as increased summer mortality rates in abalones and other ectothermic slow-crawling shellfishes [1]. For example, increases in atmospheric temperature are reflected in the decreased oxygen solubility in coastal waters, which creates hypoxic conditions for various aerobic underwater organisms, thus causing them to experience internal energy imbalances [2]. These stressed shellfishes are highly susceptible to pathogens, increasing the mortality rate in aquaculture systems [3]. In Pacific abalone (*H. discus hannai*), deviations from the optimal temperature (20 °C) resulted in suppressed lysozyme activity, reducing immune activity against bacterial infection and leading to higher mortality [4]. These scenarios are more common in coastal-based marine aquaculture systems because temperature and oxygen cannot be controlled manually, as the systems are subject to environmental conditions. In abalone physiology, in contrast to chronic thermal stress, short-term thermal stress does not influence energy metabolism [1,5,6]. In most cases, when abalone are subjected to higher temperatures for a prolonged period, their energy metabolic state shifts from aerobic to anaerobic, which also affects growth and causes weight loss due to overutilization of energy and reduction of food intake [3].

Another challenge for marine environments is global warming, which is causing great damage to corals and other marine ecosystems globally [7,8]. This is reflected in capture production, and affects the food chain of coastal indigenous peoples and other populations around the world [9]. An alternative source for marine capture is aquaculture industries, which are used to enhance sustainable production; 62% of aquaculture production is estimated to be used as human food by 2030 [10]. However, while abalone aquaculture production is limited compared to other fishes, it has huge market value worldwide. Abalone meat costs approximately $25–27 per kilogram in the United States, according to a 2016 report from the US Food and Agriculture Organization. Since the 1970s, aquaculture systems for abalone have been continuously promoted in South Korea to maintain sustainable production and meet the global market demand. South Korea has since held fourth place in overall seafood production and was the second largest producer of farmed abalone in the world in 2014 [1,11]. However, a current problem in coastal-based aquaculture is summer mortality, which leads to the highest production loss among farmers [1]. To mitigate temperature-related problems, in-depth knowledge of abalone genetics is required. In particular, for *H. discus hannai*, few de novo transcriptome profiling studies have been conducted to capture transcript expression patterns under various stresses [12,13,14] since the release of the draft genome [15]. In most studies of Pacific abalone and other abalones, heat shock family proteins were randomly selected as molecular quantification markers for thermally challenged abalones [16,17,18].

Heat shock proteins (HSPs), major components of the chaperone systems, are key regulators in the process of cellular acclimatization to various stresses and are ubiquitous in almost all multicellular organisms. The HSPs are categorized into six major families: HSP100, HSP90, HSP70, HSP60, HSP40, and small HSPs (sHSPs) [19,20,21]. HSPs also act as housekeeping genes to maintain cellular hemostasis under various stress conditions by adjusting the molecular machinery. These proteins are upregulated under various stress conditions and are not only specific to thermal stress, as they act as common machines to regulate basic cell functions. HSPs work individually and also cooperate with sHSPs to perform various molecular chaperone functions in cells [21]. Notably, the cellular HSP level can act as a potential indicator to identify the thermal stress status of abalone [22]. With this knowledge, we designed this study to systematically identify HSP gene expression patterns associated with various thermal conditions (i.e., below or above the optimal temperature) in Pacific abalone. In particular, we examined expression patterns in different tissues (i.e., hemocytes, gill, mantle, and muscle), as they react differently under different thermal conditions to protect the abalone; e.g., the gill and mantle are respiratory organs and respond differently under stress conditions compared to muscle [5]. In addition, HSPs and their cooperators (i.e., sHSPs) are the deciding factors for various housekeeping functions [21], thus we also compared the Pacific abalone proteome with those of other shellfishes and representatives from different taxa (i.e., animals, plants, insects, worms, yeast, and bacteria) to understand the homologs of HSP family proteins in Pacific abalone.

## 2. Materials and Methods

### 2.1. Pacific Abalone Sampling

**Figure 1:**
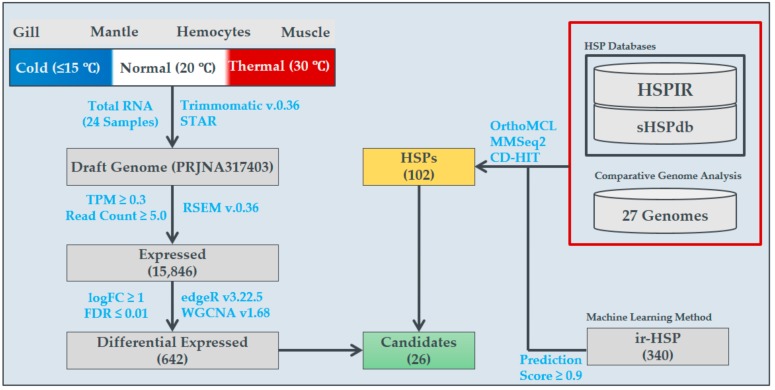
Illustration of the systematic bioinformatics analyses performed to elucidate temperature-specific gene expression in *H. discus hannai* (Pacific abalone), as well as heat shock protein (HSP) expression profiles.

Pacific abalone *H. discus hannai* (mean shell length, 54.69 ± 3.78 mm; shell width, 37.09 ± 2.63 mm; body weight, 16.44 ± 3.87 g) were purchased from Wando, South Korea, in March 2017. Abalone were maintained and reared in 1-ton tanks filled with natural seawater (12 °C) with a continuous flow system and aeration. The complete experimental procedure is illustrated in Figure 1. For the gradual temperature change, the seawater temperature was constantly increased at a rate of 1 °C/h until reaching the maximum temperature (30 °C) using an automatic aquatron system (Yoowon Electronics, Gyeonggi-do, South Korea); three animals were sampled at each experimental temperature (≤15 °C, 20 °C, and 30 °C (each temperature maintained for an hour)). During this time, the oxygen level (7.5 ± 0.5 mg/L) was properly maintained. Tissue samples (hemocytes, gill, mantle, and muscle) collected from each animal were snap-frozen in liquid nitrogen and stored at −80 °C until RNA isolation.

### 2.2. Total RNA Sequencing and Analysis

The complete library preparation procedure for RNA sequencing (RNA-Seq) on the Illumina HiSeq 2000 platform was conducted through DNALink, an authorized service provider in South Korea. The raw sequences were subjected to bioinformatics analysis, as illustrated in Figure 1. First, low quality sequences (PHRED score; Q ≤ 20) and adapter contamination were eliminated using Trimmomatic v.0.36 [23]. The cleaned sequences were mapped to the draft version of the reference genome [15] using the STAR mapper, along with the RNA-Seq by the Expectation Maximization (RSEM) method to obtain the expression value for each gene/transcript in the genome [24]. The read counts estimated by RSEM were subjected to edgeR v.3.22.5 to obtain differential expression scores and determine statistical significance [25]. Here, two samples each from low-, optimal-, and high-temperature conditions were used for sequencing to determine statistical significance and differential expression profiles among high- and low-temperature conditions in comparison to optimal-temperature conditions were obtained. Similarly, the differential expression among the tissues were also executed. Several filters, namely, transcripts per million (TPM) ≥ 0.3, read count ≥5, and log fold change ≥1, were applied for the selection of differentially expressed transcripts. Finally, the expressed transcripts (i.e., TPM ≥ 0.3 and read count ≥ 5) were subjected to co-expression network construction with weighted gene co-expression network analysis (WGCNA) [6]. The networks were constructed for individual and pooled tissues to observe the co-expression of genes/transcripts responsible during high-temperature conditions, with three sub-modules, namely, expressed genes, differentially expressed genes, and HSPs. The RNA sequences were submitted to the National Center for Biotechnology Information Sequence Read Archive (NCBI-SRA) repository under accession numbers SRR9859054–SRR9859073 and SRR10127522–SRR10127525.

### 2.3. HSP Ortholog Search

The HSP ortholog search was conducted using two different strategies, namely, alignment-based and alignment-free similarity searches to identify homologous proteins from shellfish from various habitats as well as with other representative organisms from the tree of life (i.e., animals, plants, insects, worms, yeast, and bacteria). To identify HSPs and their sub-families, the datasets were obtained from two different databases in the literature, the heat shock protein information resource (HSPIR) [20] and the small heat shock protein database (sHSPdb) [19]. Here, sequences that cluster together with the HSP database sequences were considered orthologous proteins to the specific family. For the similarity clustering search, we used two algorithms, MMseqs2 [26] and CD-HIT v.4.6 [27]; for the ortholog search we used orthoMCL v.2.0 [28]. To include all possible HSP transcripts/genes, we included the proteins from all three searches, and comparative heatmaps were plotted, as explained in [29]. In addition, due to the limitations of database updates, a machine-learning method developed to derive features for HSPs from primary sequences, ir-HSP [30], was also used to predict HSPs.

### 2.4. Experimental Validation with qRT-PCR

To validate the transcriptome dataset, several HSP genes were systematically selected for reverse transcription (RT)-PCR confirmation. The RNA samples of hemocytes, gill, mantle, and muscle were reverse transcribed using the Advantage RT-for-PCR kit (BD Biosciences, San Jose, CA, USA). Primer sequences were designed using Primer 3 the related information is shown in Table 1. Real-time (q) PCR was performed using Fast SYBR Green Master Mix (Applied Biosystems, Foster City, CA, USA) with an initial 10 min Taq enzyme activation step at 95 °C, followed by 40 cycles of 95 °C for 10 s, 60 °C for 5 s, and 72 °C for 20 s, with fluorescence reading in an SDS 7500 system (Applied Biosystems). The correlation coefficient was calculated between log fold change of relative quantification values and RNA-Seq to find the linear relationship.

### 2.5. Ethics Statement

We conducted all experiments as instructions (NIFS Instruction no. 594), which was approved by the ethical committee formed for animal experiments by the National Institute of Fisheries Science (NIFS), South Korea.

## 3. Results

### 3.1. Sequencing and Differentially Expressed Genes

**Figure 2:**
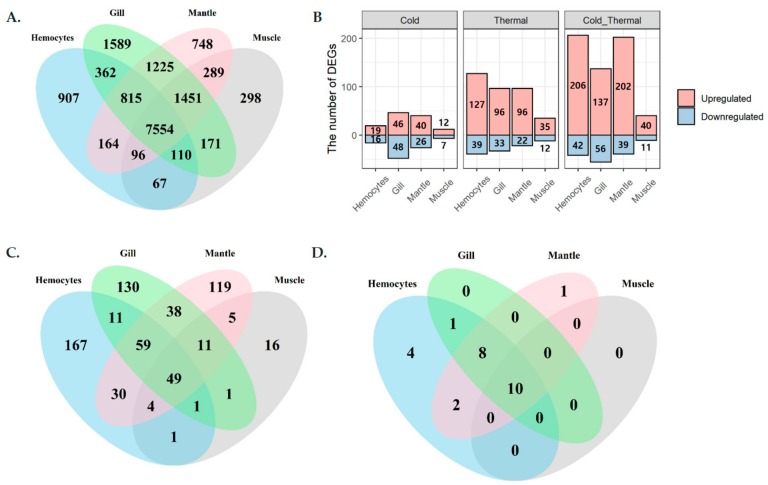
Tissue-specific expression and differential expression profiles among cold and thermal-stress conditions, observed by total RNA sequencing (RNA-Seq) analysis. (**A**) Tissue-specific expression, (**B**) differentially expressed genes among cold- and thermal-temperature conditions while compared with optimal temperature, (**C**) differentially expressed genes in different tissues, and (**D**) differentially expressed HSPs.

In total, 100.9 Gb of total RNA was sequenced from four different abalone tissues (hemocytes, gill, muscle, and mantle) and three different temperature groups (low, optimal/normal, and high/thermal) (Figure 1). Among them, 96.7 Gb remained after filtering out sequencing artifacts and 76.5 Gb were mapped to the *H. discus hannai* draft genome (Supplementary Figure S1). Of the mapped sequences, 11.9 Gb were mapped to the gene-coding region and contributed to the expression quantification of 15,846 (46.5%) genes (Figure 2A). Among these, 642 genes exhibited differential expression under cold or heat stress (Figure 2B,C), including 26 HSP genes (Figure 2D). Hemocytes exhibited the highest differential expression compared to other tissues, including four specific HSPs. So far, the heat HSPs were randomly selected for thermal condition quantifications and assessments. To overcome the random gene selection for characterizations, we conducted systematic bioinformatics analysis to identify HSP expression, tissue-specific gene expression, and their co-expression in Pacific abalone, to obtain a detailed characterization of HSPs from Pacific abalone and other shellfish that inhabit the intertidal zone.

### 3.2. HSPs

**Figure 3:**
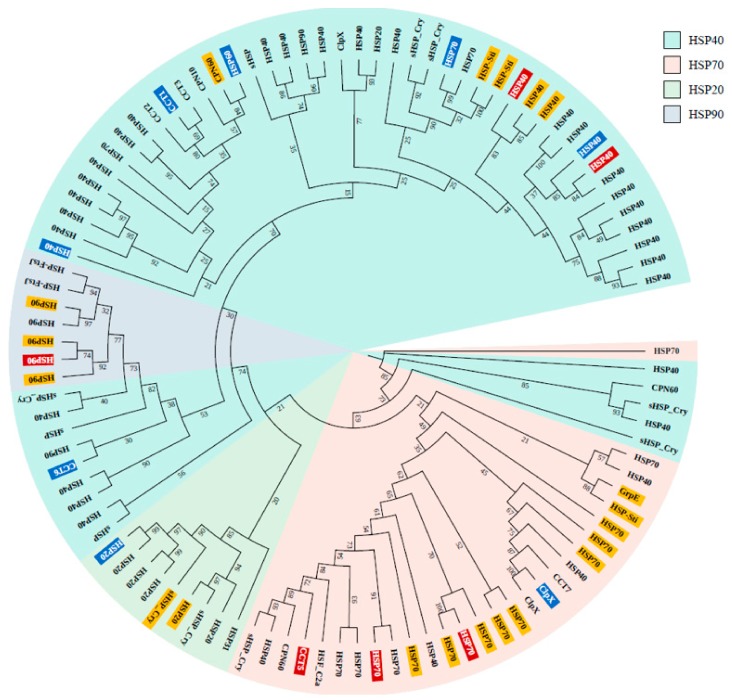
Phylogenetic tree of 102 HSPs annotated using ortholog and clustering methods (ir-HSP annotated proteins are not included). The tree was reconstructed from 1000 bootstrap replicates (bootstrap values included in their respective branches). The highlighting colors indicate HSPs selected for qRT-PCR (blue), differentially expressed HSPs (yellow), and differentially expressed HSPs selected for qRT-PCR (red).

In total, 413 HSPs were identified from three different sequence similarity search methodologies (OrthoMCL, Linclust, and CD-HIT) developed to identify homologous proteins (Supplementary Figure S2), as well as one machine-learning model that derives the features from primary protein sequences to predict HSPs. The ir-HSP machine-learning model provided more HSP annotations compared with the sequence similarity-based approaches. Among the sequence similarity approaches, the OrthoMCL method contributed more HSP annotations (Supplementary Figure S2). Overall, 15 proteins were annotated as HSPs from the four methods. The proteins annotated as HSPs from the sequence similarity approaches were further added to a phylogenetic tree to confirm the HSP family classification. Most of the annotated HSPs belonged to the HSP40 super family, followed by the HSP70, HSP90, and HSP20 families (Figure 3).

**Figure 4:**
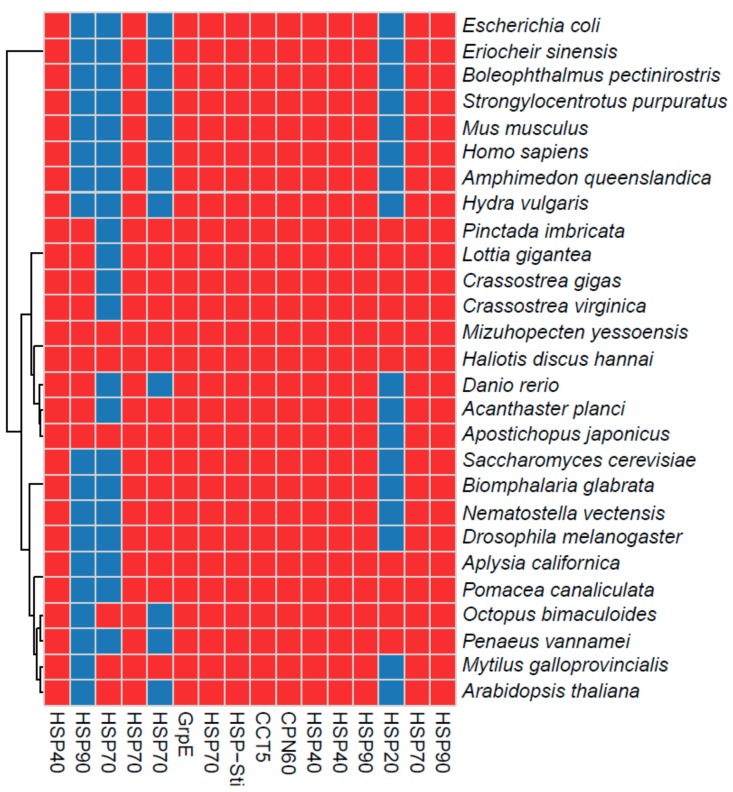
Differentially expressed HSPs in *H. discuss hannai* and comparative profiles with other shellfish inhabiting intertidal zones, as well as humans, plants, insects, yeast, and bacteria. Red and blue denote the presence of *discus hannai* and absence of the homologous proteins, respectively. The binary values were obtained using the orthoMCL method.

This phylogenetic tree was used to select HSP candidates for qRT-PCR. Finally, the HSPs were compared to those from other species that inhabit intertidal zones, including species with different phenotypes, ranging from soft-bodied to shelled organisms. We observed that most of the HSPs were ubiquitous to most of the selected species, although several proteins were limited to only a few species; e.g., HSP20 in the heat map (Figure 4). These comparative profiles and detailed annotations of the HSP super families will provide a good resource for future studies.

### 3.3. HSP Expression and Co-Expression Profiles

**Figure 5:**
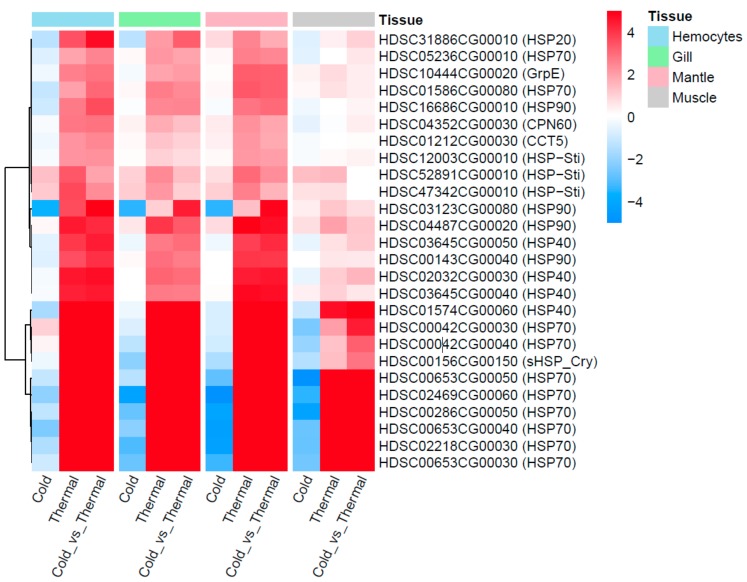
Twenty-six differentially expressed HSPs observed from four different tissues and three temperature conditions (cold, optimal and thermal).

**Figure 6:**
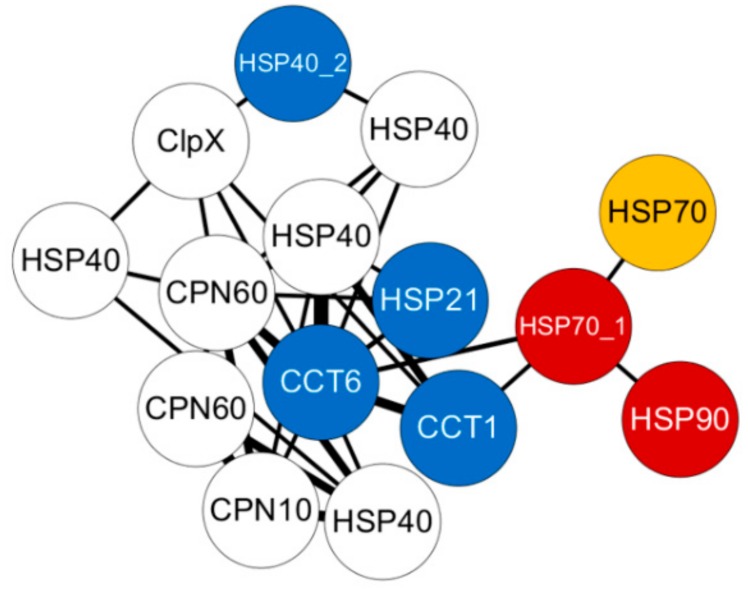
Weighted gene co-expression network analysis (WGCNA) co-expression network derived from Module 3, which only includes annotated HSP genes. The sub-network of turquoise, from gill tissue was plotted using Cytoscape, with edge weight ≥0.2. Colored circles indicate differentially expressed genes (yellow), genes selected for qRT-PCR (blue), and differentially expressed genes selected for qRT-PCR (red).

**Figure 7:**
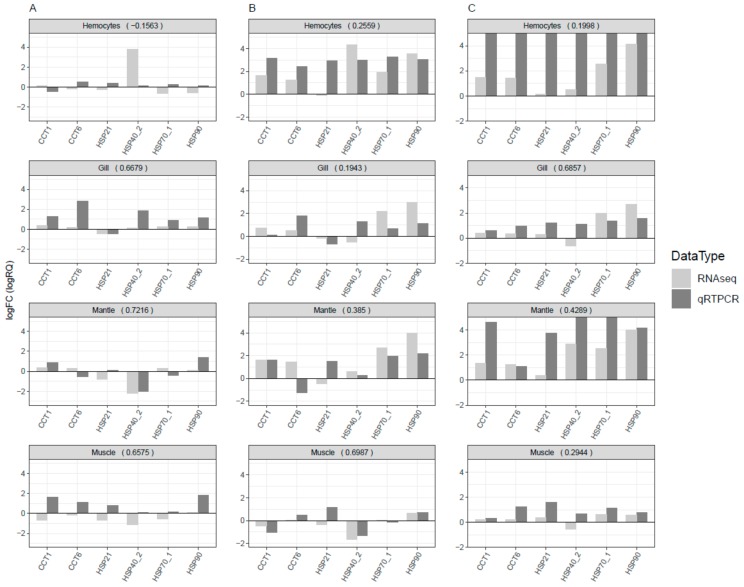
Systematically selected HSP genes for qRT-PCR validation to assess the reproducibility of the RNA-Seq expression profiles from different experimental replicates. The log fold change values from both experiments were compared and Pearson correlation coefficients were calculated to assess the linear relationship between the experiments. The log fold change values for RNA-Seq were obtained from transcripts per million, and relative quantification values were used from qRT-PCR. Values >5 were reduced to 5 to enhance the visibility of the bar chart and present a common scale for easy interpretation. (**A**) Optimal vs. cold; (**B**) optimal vs. thermal; and (**C**) thermal vs. cold.

Twenty-six HSPs were observed with differential expression under cold and thermal-stress conditions. Among them, ten HSPs were expressed in all four tissues, hemocytes had four tissue-specific expressions, and gill tissue had the highest number of HSPs compared to other tissues (Figure 2D). Most of the proteins annotated as HSP70 were highly expressed under the thermal-stress condition and suppressed under the cold condition (Figure 5). The co-expression patterns of expressed HSPs (HSP40, HSP20, and HSP90) observed in gill tissue are illustrated in Figure 6, demonstrating the chaperone function of the protein machinery, which was exponentially elevated in abalone under thermal stress. The genes annotated as HSP70 were highly co-expressed in all four tissues (Supplementary Figure S3). Multiple gene isoforms of each HSP family are present in the Pacific abalone genome; representative proteins were selected from different branches of the phylogenetic tree (Figure 3) and validated by qRT-PCR to assess the significance of the expression patterns (Figure 7). In total, 14 proteins were randomly selected from the annotated HSP family genes, six of which were highly co-expressed in the gill and responsive to thermal stress (Figure 5 and Figure 7; Supplementary Figure S4). The expression patterns observed for HSP70 and HSP90 isoforms under thermal stress were similar in both qRT-PCR and RNA-Seq, but the half of the randomly selected transcripts did not show similar expression patterns in the two analyses.

## 4. Discussion

The primarily challenge for every living organism on the Earth is to optimize energy requirements under various environmental conditions so as to perform basic physiological processes. Interestingly, the broadcast-spawning physiological nature of abalone facilitates the molecular switch between two states; i.e., aerobiosis and anaerobiosis to perform the basic cellular functions at various thermal conditions [3,5]. This was demonstrated by the positive correlation between temperature and oxygen solubility, and the negative correlation between oxygen solubility and metabolic rate observed in abalone under thermal stress [3,13]. Generally, the growth and reproduction of abalone also differed under thermal and cold temperatures in comparison to optimal-temperature (20 °C) conditions. In particular, abalone mortality was higher under chronic thermal-temperature conditions compared to cold conditions [17]. To understand these cellular processes with respect to the transcriptome, we conducted this study to observe gene/transcript expression patterns in Pacific abalone tissues.

Under thermal stress, a ubiquitous function of cells was the overproduction of reactive oxygen species (ROS), followed by increased levels of antioxidants as well as HSPs, which degrade the damaged proteins [3,4]. The initial defense against thermal stress began with antioxidants; if ROS levels are still high after inhibition by antioxidants, HSPs are activated to reduce ROS levels and prevent cell death. Based on this principle, most studies in abalone randomly selected HSP70 as a molecular marker to assess the thermal condition. However, the release of the draft genome [15] facilitated the analysis of transcriptome-wide expression profiles in a short time span.

In this study, we examined tissue-specific gene expression profiles from four different tissues as well as differential expression profiles among thermal- and cold-temperature conditions in comparison to optimal-temperature conditions for highly expressed genes in Pacific abalone (Supplementary Table S1). Furthermore, using in-house libraries, we obtained reliable co-expression networks, which were constructed into three sub-modules, namely, expressed, differentially expressed, and within HSPs, extending the known functional connectivity among these highly expressed genes [31]. For example, HSPs were highly cooperative with each other to perform molecular chaperone functions; in particular, HSP70 and HSP90 cooperated in protein folding machineries along with other sHSP co-chaperones to regulate energy metabolism [21]. Similarly, in our results, the networks constructed individually for each tissue using only the differentially expressed genes illustrated the strong co-expression of HSP70 genes and the sHSP HSP26 (Supplementary Figure S3). In Figure 6, in the network derived from the gill constructed only using annotated HSPs; CCT1, CCT6, HSP70, HSP90, and HSP40 were highly co-expressed along with other molecular chaperones to enhance energy normalization in Pacific abalone under thermal stress. A similar approach was deployed in *Haliotis fulgens* to explore acute hypoxia and hypercapnia under thermal-stress conditions using a de novo transcriptome, and a co-expression network was constructed among the targeted genes [18]. Previously in Pacific abalone, the gene expression of HSP70 and HSP40 was reported to vary among thermal-sensitive and thermal-tolerant abalones, and the thermal-tolerant line showed higher expression levels than the thermal-sensitive lines [13]. In *Haliotis diversicolor*, HSP70 was highly expressed in hemocytes under both thermal and pathogen stress due to activation of the immune response [32,33]. Similarly, HSP90 was more sensitive to acute thermal stress then to chronic thermal stress, which was observed in the gill of *Haliotis discus* [16,17], while HSP70 was observed to be sensitive to acute and chronic thermal stress in *Haliotis rufescens* [33,34]. Among the sHSPs, HSP20 and HSP26 were significantly expressed in muscle and mantle tissue of *H. discus* under thermal stress [22,35].

Tissue-specific RNA-Seq profiling can help identify the unique roles of genes in different tissues. In our results, the highest gene expression levels were observed in the gill, a primary organ that plays a major role in habitat adaptation, waste excretion, and oxygen transport. Notably, HSP expression in hemocytes was reproducible, as most of the candidates selected for qRT-PCR analysis showed similar expression patterns in both experiments (Figure 7 and Supplementary Figure S4). Furthermore, in Pacific abalone, 55% of the body is composed of hemolymph, suggesting that hemocytes may function as a standard tissue to assess stress status in comparison to other tissues [13].

Finally, the comparative HSP profiles from intertidal organisms provide a resource for future characterizations of HSPs and explorations of common molecular functions in shellfishes. Previously from abalone, only the HSP70 family was assessed in detail in *Haliotis laevigata*, and this was also with a de novo transcriptome [36]. Although the present study was conducted using a draft genome, some limitations remain. For example, because of the use of a whole RNA-Seq protocol to generate libraries, we could only quantify 46.5% of Pacific abalone genes. Despite this limitation, the prepared dataset along with the HSP expression profiles will be a good resource for conducting meta-analyses and enhancing the characterization of the abalone draft genome. All of the expression data and annotations are included in Supplementary Table S1.

## Figures and Tables

**Table 1:** Primer sets for genes analyzed by qRT-PCR.

Primer Name	Forward Primer (5′→3′)	Reverse Primer (5′→3′)	Accession No.
CCT1	CCAAGACTGTCGTCGTTGGA	ATCTGGTGGCCAAACTACGG	HDSC01672CG00010
CCT5	CTGAAGACTTCTCTGGGCCC	GTTGTTGTGCTAGCAGGTGC	HDSC01212CG00030
CCT6	GCCTCCCTGATTGCAAGAGT	AGGTGAAGATTGAGCGCACA	HDSC00247CG00020
ClpX	AGTCTGTCGGAGGAGATGCT	CGCGCTATCATGGAGTCCAT	HDSC00459CG00020
HSP40_1	AACACGGAAACTGGCTTTGC	TGTCGAGGTCAAGGGGAGAT	HDSC02032CG00030
HSP40_2	AAGTGCAAGCGACGGTGATA	GGAGGGACTGAAGAACGGTG	HDSC05440CG00010
HSP40_3	GAAGGTGTCCCTGGTGAAGG	CAGGAAGAGGAAGTGCTGGG	HDSC00041CG00070
HSP70_1	CCTGTTCCGTTCCACCATGA	TCAACCCTGACGAAGCTGTC	HDSC05236CG00010
HSP21	CGGGTTCACATTCCTGCTCT	TTGTCACTAACGAGGACGGC	HDSC01558CG00030
HSP40_4	CCCGGAAAGTTCTCAACCCA	CAAACCCCACGCTCAGTTTG	HDSC01574CG00060
HSP60	GTGGTCGAGAAGGTGCTTCA	AGGCGGTCGTCACAGAAATT	HDSC14552CG00010
HSP70_2	ATGAAGGCGAGAGAGCGATG	ACGACAAGGGAAGGCTAAGC	HDSC00042CG00040
HSP70_3	AAAGAAGGTCCGTTGCACGA	GAGTTCGTCACGGCCTACAT	HDSC30216CG00010
HSP90	TTGCAGAGAGGGTGGTTGTC	GGAGCGTCGTATCAAGGAGG	HDSC00143CG00040

## Supplementary Materials

The following are available online at https://www.mdpi.com/2073-4425/11/1/22/s1. Figure S1. Overview of the sequencing, preprocessing, and mapping statics of the completed samples. Figure S2. Overview of the annotated HSPs from the four methods. Figure S3. WGCNA co-expression network constructed with differentially expressed genes (Module 2). Figure S4: Selected HSP genes for qRT-PCR validation to assess the reproducibility of the RNA-Seq expression profiles from different experimental replicates. Table S1. Details of expression and co-expression values along with all the annotations.
